# Vascularization of Air Sinuses and Fat Bodies in the Head of the Bottlenose Dolphin (*Tursiops truncatus*): Morphological Implications on Physiology

**DOI:** 10.3389/fphys.2012.00243

**Published:** 2012-07-04

**Authors:** Alex Costidis, Sentiel A. Rommel

**Affiliations:** ^1^Aquatic Animal Health Program, Department of Physiological Sciences, College of Veterinary Medicine, University of FloridaGainesville, FL, USA; ^2^Biology and Marine Biology, University of North Carolina WilmingtonWilmington, NC, USA

**Keywords:** delphinid, plexus, pterygoid, sinus, veins, fat, decompression sickness

## Abstract

Cetaceans have long been considered capable of limiting diving-induced nitrogen absorption and subsequent decompression sickness through a series of behavioral, anatomical, and physiological adaptations. Recent studies however suggest that in some situations these adaptive mechanisms might be overcome, resulting in lethal and sublethal injuries. Perhaps most relevant to this discussion is the finding of intravascular gas and fat emboli in mass-stranded beaked whales. Although the source of the gas emboli has as yet to been ascertained, preliminary findings suggest nitrogen is the primary component. Since nitrogen gas embolus formation in divers is linked to nitrogen saturation, it seems premature to dismiss similar pathogenic mechanisms in breath-hold diving cetaceans. Due to the various anatomical adaptations in cetacean lungs, the pulmonary system is thought of as an unlikely site of significant nitrogen absorption. The accessory sinus system on the ventral head of odontocete cetaceans contains a sizeable volume of air that is exposed to the changing hydrostatic pressures during a dive, and is intimately associated with vasculature potentially capable of absorbing nitrogen through its walls. The source of the fat emboli has also remained elusive. Most mammalian fat deposits are considered poorly vascularized and therefore unlikely sites of intravascular introduction of lipid, although cetacean blubber may not be as poorly vascularized as previously thought. We present new data on the vasculature of air sinuses and acoustic fat bodies in the head of bottlenose dolphins and compare it to published accounts. We show that the mandibular fat bodies and accessory sinus system are associated with extensive venous plexuses and suggest potential physiological and pathological implications.

## Introduction

Since the 1980s, numerous beaked whale mass strandings have been temporally and/or spatially associated with deployment of naval mid-frequency active sonar (Simmonds and Lopez-Jurado, 1991; Frantzis, 1998; Evans and England, 2001; Fernandez et al., 2004, 2005). Research into potential causal mechanisms underlying these events is logistically difficult, in part because of constraints such as insufficient funding and the cryptic nature of beaked whales and because of legal and public constraints surrounding live-animal physiological experimentation (reviewed in Cox et al., 2006). Most of the progress that has been made on this topic has been made either through tagging of live beaked whales (Tyack et al., 2006) or from post-mortem morphological and pathological studies (Fernandez et al., 2004, 2005; Hooker et al., 2009). Hypotheses regarding the etiology of the strandings range from physical acoustic trauma to systemic gas and fat embolic syndrome caused by behavioral alterations in dive profiles (Cox et al., 2006; Rommel et al., 2006; Hooker et al., 2011). The tagging studies have generated invaluable insights into the diving behavior of certain beaked whale species, and these data have provided a platform for modeling of gas kinetics such as nitrogen uptake and elimination (Houser et al., 2001; Fahlman et al., 2006; Tyack et al., 2006; Zimmer and Tyack, 2007; Hooker et al., 2009).

Post-mortem examinations of beaked whales stranded in the Canary Islands have identified decompression sickness (DCS)-type sequelae, suggesting that gas bubble formation may be at the root of some of the observed strandings (Fernandez et al., 2004, 2005). Additional findings of apparent acute and chronic embolization in other odontocete species suggest that gas bubble disease in cetaceans may be more common than initially thought (Jepson et al., 2003, 2005). Much debate has focused on individual physiological, behavioral, or anatomical traits that may predispose beaked whales to nitrogen saturation and subsequent DCS-like lesions (Cox et al., 2006; Fahlman et al., 2006, 2009; Rommel et al., 2006; Hooker et al., 2009). Following Scholander’s legacy of comprehensive consideration of all aspects of diving biology, we suggest that a combination of physiological, anatomical, and behavioral characteristics contribute to this predisposition and that research should focus on integrating all the aforementioned characteristics (Scholander, 1940).

**Figure 1:**
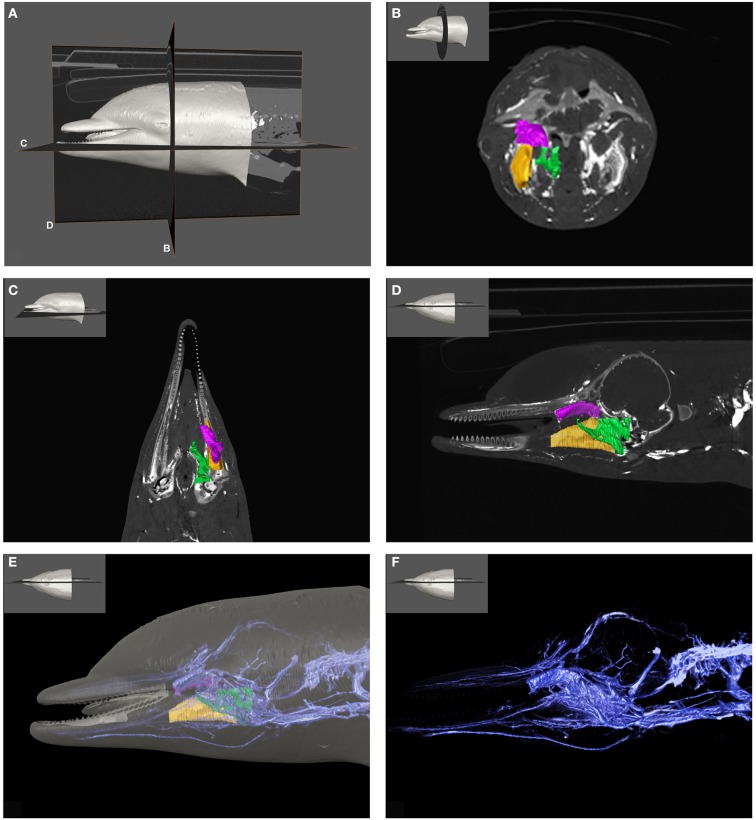
**Plate combining computed tomographic (CT) slices with volume renderings of the pterygoid and peribullar plexus (green), intramandibular fat body plexus (yellow), and anterior lobe (purple) to illustrate overall location of the structures**. Bright white areas in CT slices, and dark blue structures in volume renderings represent contrast enhanced venous structures. **(A)** Shows the plane of section for **(B–D)**. **(B)** Shows a transverse view, **(C)** shows a coronal view, and **(D)** shows a sagittal view. **(D)** Shows a reference outline of the external surface of the dolphin overlaid on a three-dimensional reconstruction of the venous system (blue) and structures of interest. **(E)** Shows only the venous system so the more detailed structures can be observed. **(F)** Shows a medial view of the veins of the right side of the head, as seen from a mid-sagittal plane of section. Note that the small inset panels within each larger panel show the orientation of the plane of section for each panel.

Currently, our only insights into the functional implications of the anatomy are to be inferred from pathological findings. To begin this discussion, we chose to focus on the lesions that were observed in the beaked whales that stranded in the Canary Islands in September of 2002 (Fernandez et al., 2005). The most conspicuous lesions discovered on post-mortem examination were intravascular emboli widely disseminated throughout the kidneys, lungs, liver, and central nervous system. Interestingly, these emboli were found to sometimes be composed of gas, other times of fat; however both were often present simultaneously within a single tissue. These findings suggest that either different body areas or types of tissue were compromised separately or a single region containing intimate gas and fat associations was damaged. In either situation, in addition to the involvement of gas-filled spaces and fatty tissues, the other requirement for such dissemination of the emboli is vascular introduction and transportation. Therefore, unless there was intravascular introduction of gas and fat throughout the body or generalized autochthonous embolus formation, we suggest that the most likely location for vascular introduction of gas and fat emboli would be anatomical regions where gas, fat, and blood vessels are intimately associated (Figure 1; see also Movie S1 in Supplementary Material). We therefore agree with Jepson et al. (2005) who alluded to the fatty tissues of the odontocete head as a reasonable source of fat emboli due to the extensive vascular structures associated with both cranial air-filled sinuses and acoustic fat bodies and the frequent observation of hemorrhages within those tissues in DCS-like cases of strandings (Boenninghaus, 1904; Slijper, 1936; Fraser and Purves, 1960; Ridgway et al., 1974; McFarland et al., 1979; Fernandez et al., 2004, 2005).

There is a relative paucity of published information concerning beaked whale anatomy, especially the anatomy relating to diving physiology or issues of potential susceptibility to mid-frequency active sonar (reviewed in Rommel et al., 2006). Gas kinetic modeling has shown that knowledge of the vascular anatomy is integral to understanding the dynamics of nitrogen gas uptake and elimination (Fahlman et al., 2006, 2009; Hooker et al., 2009). Some of the most relevant beaked whale anatomy relating to diving physiology is therefore, likely to be the morphology and function of blood vessels, about which nothing has been published. Although the arterial system of cetaceans has received considerable attention, the venous system has remained largely undescribed (Breschet, 1836; Murie, 1873; Wilson, 1879; Ommanney, 1932; Slijper, 1936; Walmsley, 1938; Harrison and Tomlinson, 1956; Fraser and Purves, 1960; Viamonte et al., 1968; Du Boulay and Verity, 1973; McFarland et al., 1979; Vogl and Fisher, 1981, 1982; Vogl et al., 1981). Given the lack of information on beaked whale vascular anatomy, this work will explore the vasculature associated with air spaces and fat bodies of the bottlenose dolphin head as it might relate to the formation of gas and fat emboli and the absorption and elimination of nitrogen gas. We hope that the information contained herein acts like a springboard from which our knowledge of the vascular anatomy of deep diving cetaceans can evolve.

**Figure 2:**
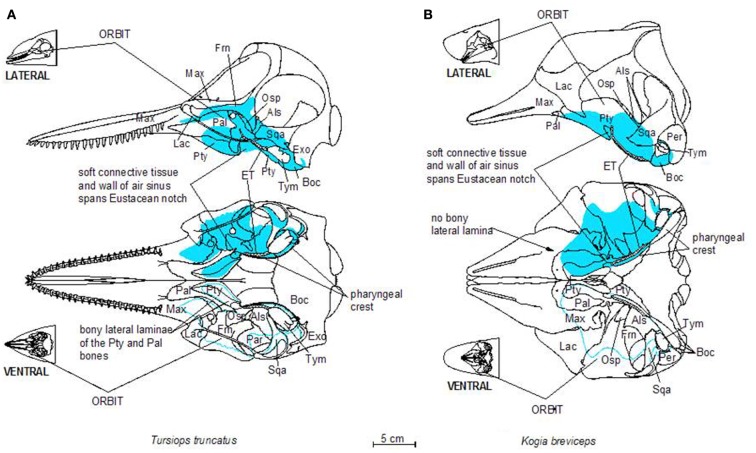
**Schematic illustration of dorsal and ventral skeletal associations to the accessory sinus system in a bottlenose dolphin (A) and a pygmy sperm whale (B)**. Three letter abbreviations refer to bone names as follows: Als, alisphenoid; Boc, basioccipital; Exo, exoccipital; Frn, frontal; Lac, lacrimal; Mas, mastoid (mastoid process of the periotic bone); Max, maxilla; Osp, orbitosphenoid; Pal, palatine; Pty, pterygoid; Sqa, squamosal; Tym, tympanic bulla; Vom, vomer.

**Figure 3:**
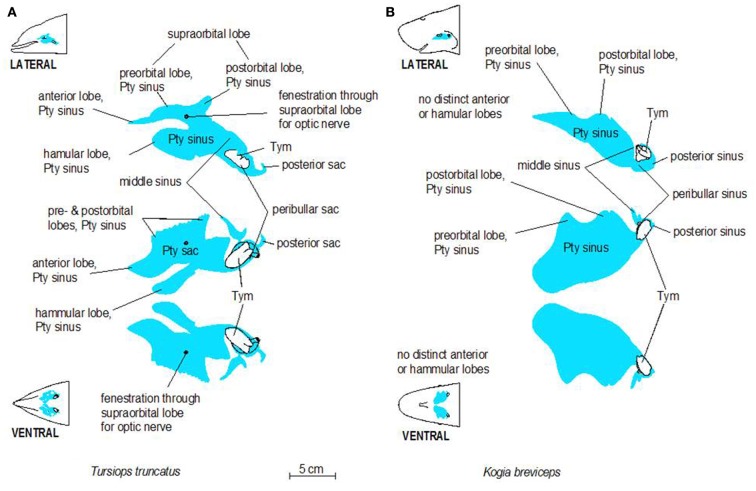
**Schematic illustrations of lateral and ventral views of the pterygoid sinus system in bottlenose dolphins (A) and pygmy sperm whales (B)**. Note the more complex lobes observed in dolphins, relative to the simple geometry of the system in pygmy sperm whales.

Before discussing the vascular anatomy of the region, a presentation of anatomical details of the accessory sinus system seems prudent. Cetaceans have lost the air-filled paranasal sinus system found in terrestrial mammals (Mead, 1975; Mead and Fordyce, 2009). However, cetaceans do have gas-filled sinuses (the gas is derived from respiratory air but may vary in composition) that are similar to those of the paranasal system. The cetacean accessory sinus system (Figures 2 and 3) is unique (Fraser and Purves, 1960; Mead and Fordyce, 2009); these un-pigmented mucosa-lined structures, which are located on the ventral aspect of the skull, are typically associated with hearing and acoustic isolation of the ears (Houser et al., 2004). The ventral sinus system is distinguished from the dorsal air sacs by appearance and function; the lining of the dorsal sacs is composed of pigmented epithelium (Reidenberg and Laitman, 2008) and these sacs are associated with sound production. Both sets of air-filled structures are confluent with the respiratory system.

In their stunning monograph, Fraser and Purves (1960) outlined much of the anatomy of the pterygoid and peribullar sinuses and their surrounding auditory structures by piecing together information from corrosion casts and dissections of various delphinid species and compiling published literature. Their anatomical descriptions included the bones, sinuses, and vascular structures of the region and have acted as the foundation of our review and much of our research on this topic as a whole. Fraser and Purves (1960) noted the extensive vascular investment of the lining of the accessory sinus system present in delphinid species. The vascular nature of the pterygoid and peribullar tissues surrounding and investing these sinuses has been highlighted in other cetaceans, including mysticetes such as fin whales (Murie, 1873; Boenninghaus, 1904; Ommanney, 1932; Walmsley, 1938).

**Figure 4:**
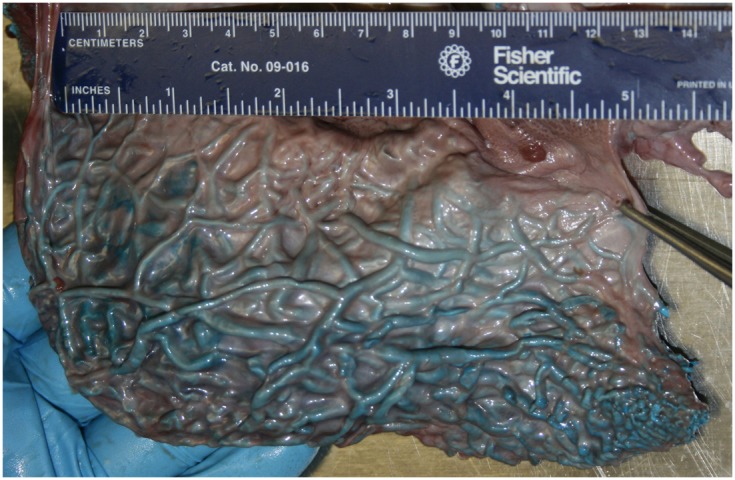
**Medial view of the pterygoid venous plexus that lines the lateral wall of the pterygoid sinus of a neonatal sperm whale (*Physeter macrocephalus*)**. Note the considerable volume and complexity, and the very thin wall separating the venous blood from the sinus air.

The accessory sinus system is therefore composed of a set of interconnected and physiologically dynamic structures (Figure 3) that have been described as both gas-filled and blood-filled (Fraser and Purves, 1960). This apparent contradiction can be explained because the spaces contain a series of gas-filled sinuses, the walls of which possess extensive vascular structures (Figure 4). Within this system the relative volumes of gas and blood can apparently be dynamically altered by hydraulic pressures of the respiratory system and the environment and by the amount of blood in the surrounding vascular structures (Fraser and Purves, 1960). Changes in volume of the gas-filled sinuses can be compensated for by corresponding changes in the adjacent soft tissues and with an incursion of blood if the sinus geometry is constrained by bone [Fraser and Purves, 1960; as in the hamular lobe of the pterygoid sinus (Pty sinus) – see below]. The sinuses occasionally contain a considerable amount of viscous, stable foam whose source and composition remain elusive.

The gas-filled part of the accessory sinus system is an extension of the middle ear cavity (Mead and Fordyce, 2009) that is connected to the upper respiratory system via the Eustachian tube (ET, auditory tube; Figure 2). The blood-filled portion (individual elements are often referred to as fibro venous plexuses) is an extensive network of veins and venous blood sinuses (Fraser and Purves, 1960) that we describe in detail for the first time. Fraser and Purves (1960) describe a long history of anatomical interest in the accessory sinus system, starting with descriptions of the peribullary sinus by Major in 1672 and Tyson in 1680. This system has been referred to by a variety of names such as: the pterygoid sinus system (Fraser and Purves, 1960; Mead and Fordyce, 2009); pneumatic cavities (Boenninghaus, 1904); Eustachian system (Anderson, 1978); Eustachian sacs (Fraser and Purves, 1960); postpalatine sinus (Flower, 1867); sinus cavities (Houser et al., 2004); air-filled sinuses (Cranford et al., 2008a); air sac system (Reidenberg and Laitman, 2008). Recently, the accessory sinus system and the nasal cavity were imaged in two live bottlenose dolphins (*Tursiops truncatus)* by Houser et al. (2004). The nomenclature that we use for the accessory sinus system originated with Beauregard (1894); it was refined by Fraser and Purves (1960) and clarified by Mead and Fordyce (2009). Although they are an invaluable resource, Fraser and Purves (1960) create considerable confusion with their “interchangeable use of cavity, lobe, sac, and sinus” (Mead and Fordyce, 2009).

## Materials and Methods

Specimens for this study were obtained from 24 deceased, stranded cetaceans recovered along the east coast of the United States and west coast of Florida (Table 1). Eleven bottlenose dolphins and 13 deep diving odontocetes were examined. Beach-cast carcasses were recovered by marine mammal stranding networks authorized by stranding agreements from the National Marine Fisheries Service U.S. Marine Mammal Health and Stranding Program. All work was conducted under a parts authorization from National Marine Fisheries Service, pursuant to 50 CFR 216.22 and 216.37, and with prior approval from University of Florida’s IACUC (Permit #: 200801345) and University of North Carolina Wilmington’s IACUC (Permit #: A0809-019).

**Table 1:** **List of specimens used for this study, including species, common name, total body length (TBL), date of stranding, and type of use for this research**.

Specimen ID	Species	Common name	TBL (cm)	Gender	Date stranded	Research use
CMA1109	*T. truncatus*	Bottlenose dolphin	191	F	17-May-11	A, V
Hubbs0909	*T. truncatus*	Bottlenose dolphin	249		27-Feb-09	A, V
ECW-005	*T. truncatus*	Bottlenose dolphin	284	M	16-Dec-10	A, V
MMC-Tt-0708	*T. truncatus*	Bottlenose dolphin	256	M	25-Jul-08	V
MMC-Tt-0107	*T. truncatus*	Bottlenose dolphin	271	F	12-Nov-06	I
BRF 164	*T. truncatus*	Bottlenose dolphin	262	F	23-Jul-07	V, S
RJM 003	*T. truncatus*	Bottlenose dolphin	188	F	28-Jun-08	V, S
VMSM 20031104	*T. truncatus*	Bottlenose dolphin	204	M	26-Dec-03	S
VAQS 20061067	*T. truncatus*	Bottlenose dolphin	174.2	M	6-Aug-06	V,S
PBN 003	*T. truncatus*	Bottlenose dolphin	246	F	14-Feb-08	V,S
VAQS 20051086	*T. truncatus*	Bottlenose dolphin	195.4	M	17-Jul-05	S
MML0802	*F. attenuata*	Pigmy killer whale	208	M	30-Jun-05	A, V
MMC-Pm-0908	*P. macrocephalus*	Sperm whale	324	F	29-Sep-08	A, V
VAQS 2008 1002	*K. sima*	Dwarf sperm whale	160	M	28-Jan-08	S
FMMSN0906	*K. breviceps*	Pygmy sperm whale	167	M	10-Jul-09	A, V
VAQS 20071006	*K. breviceps*	Pygmy sperm whale	263	F	27-Feb-07	V,S
CLP 001	*K. breviceps*	Pygmy sperm whale	225	M	22-Nov-07	V,S
KLC 059	*K. breviceps*	Pygmy sperm whale	223.5	M	16-Nov-09	S
KLC 025	*K. breviceps*	Pygmy sperm whale	213	F	22-Dec-08	V,S
MDB 056	*K. breviceps*	Pygmy sperm whale	263.5	M	15-Dec-09	S
MARS0903	*M. europaeus*	Gervais’ beaked whale	229	M	2-Aug-09	A, V
WAM 593	*M. densirostris*	Blaineville’s beaked whale	423	M	28-Jan-04	S
MDB 023	*M. densirostris*	Blaineville’s beaked whale	434	F	15-Sep-08	S
VAQS 20091107	*M. bidens*	Sowerby’s beaked whale	397	M	8-Nov-09	S

*Abbreviations for genus name are as follows: *T*., *Tursiops*; *F*., *Feresa*; *P*., *Physeter*; *K*., *Kogia*; *M*., *Mesoplodon*. Abbreviations for research use are as follows: A, angiography; I, MRI/CT imaging; V, vascular dissection; S, sinus dissection*.

Specimens used for gross dissection of the accessory sinus system were either dissected fresh, or frozen, thawed, and dissected when logistics allowed. Schematic illustrations of the accessory sinus system and the simplified venous connections were made using EasyCAD (Evolution Computing, Phoenix Arizona 85020). Specimens used for describing the vascular anatomy were injected with contrast medium into either the venous system or both the venous and arterial systems, following the procedural methodology outlined by Holliday et al. (2006). Prior to injection of contrast media, all specimens obtained a vascular flush using 0.9% phosphate buffered saline solution and some received a subsequent 5% neutral buffered formalin vascular perfusion to enable a protracted dissecting period. Most specimens were targeted for examination of the venous system, and were therefore flushed through the arteries and out of the veins. Once the effluent ran clear, the flush was stopped and the specimens were refrigerated while being allowed to drain for several hours.

Prior to injecting the vascular contrast material, 5 mL balloon catheters were placed in the vessels to be injected and inflated until a good seal was formed. In specimens that were to be imaged via computed tomography (CT), the vascular system of interest received a mixture of liquid latex and barium sulfate suspension (Liquid Polibar Plus, Bracco Diagnostics Inc.), while vessels only destined for dissection received pure latex (Carolina Biological Inc.). All specimens were refrigerated for 2 days following injections, to allow the latex cast to cure, and if unpreserved with formalin, where subsequently frozen at −20°C. Specimens that were imaged via CT were scanned at the thinnest slice thickness possible based on the specimen length, and whenever possible the volumes were reconstructed to 0.5 mm thickness to allow high resolution imaging of fine caliber vasculature. The resultant DICOM data was post-processed using Amira^®^ software (Visage Imaging Inc., San Diego, CA, USA) on a Gateway desktop with memory and processor upgrades. Following imaging and post-processing, all specimens were thawed and dissected to validate and/or clarify imaged structures. Although the focus of this study was to elucidate the venous morphology in regions of interest in the head of *Tursiops*, the authors felt that a comparative examination of deep diving odontocete cetaceans would be valuable given the association of deep divers and sonar-related strandings. The authors therefore opportunistically obtained specimens from pygmy and dwarf sperm whales (*Kogia, breviceps*, and *sima*), sperm whales (*Physeter macrocephalus*), and Gervais’ beaked whales (*Mesoplodon europaeus*). Some of the sperm whale and pygmy sperm whale specimens were of sufficient quality and from young enough animals that could fit into the CT gantry. Prior to vascular dissection, those specimens were imaged according to the aforementioned angiographic protocol and the data obtained was used to guide the dissections. As the focus of this study was on *Tursiops*, only cursory mention is made with respect to findings from the other species.

## Results^1^

### Air sinus and fat body morphology

#### Sinuses

**Figure 5:**
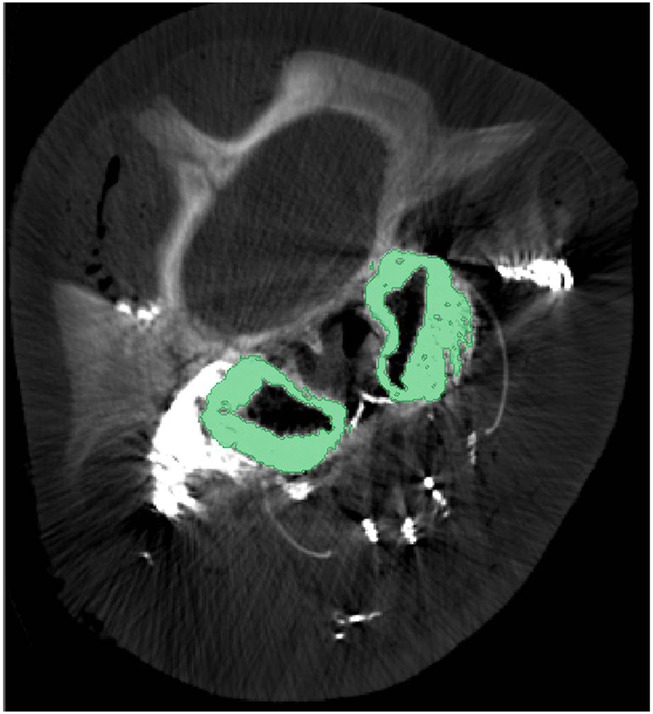
**Cross-sectional computed tomographic image at the level of the eyes of a pygmy sperm whale with contrast enhanced veins**. The green regions represent the pterygoid venous plexus that surrounds much of the pterygoid sinus. Note that during post-mortem examination the pterygoid venous plexus is usually empty and the green-colored space being occupied by it in this image is occupied by air in the expanded air sinus.

What is known about the accessory sinus system of the cetacean head varies considerably by species, however delphinid and phocoenid species remain the best described. It is however clear that in all odontocete species there are extensive gas-filled sinuses on the ventral side of the skull. Interestingly, our preliminary research shows that the accessory air sinuses of deep diving odontocetes such as beaked whales, spermwhales, and pygmy and dwarf sperm whales are much larger, relatively, than those of even large delphinid species like pilot whales, and are invested with intricate and seemingly more voluminous venous arrangements. Much of the internal surface of these sinuses is lined with copious masses of convoluted, intercommunicating, and valve-less veins separated from the air spaces by walls so thin that they are translucent (Figures 4 and 5).

### Air sinus extent

The accessory sinus system is mucosa-lined and juxtaposed (on at least one side) to bones of the basicranium. The sinus system exists as a bilaterally paired system of blind structures. These sinuses (Figure 3) may extend rostrally to cover the ventral aspect of the palate, parts of the orbit, and pharynx and caudally to surround the tympanic bulla of the ear (Tym, Figure 2), the temporomandibular joint (TMJ), and the joint between the skull and the hyoid apparatus. The accessory sinus system is dominated by the Pty sinus. This sinus extends from just rostral to the orbit back to the region of the Tym. The Pty sinus has two more or less distinct lateral lobes^1^ that may extend dorsad around the eyestalk (preorbital and postorbital lobes). In *Tursiops*, these lobes are expanded and may meet dorsal to the eye as a supraorbital lobe. The presence of an air-filled sinus on the ventral aspect of the supraorbital process of the frontal bone dorsal to the eye necessitates a fenestration (optic infundibulum; Fraser and Purves, 1960) through the sinus to accommodate passage of the optic nerve, because the sinus extends over the region where the nerve exits the braincase.

The Pty sinus in *Tursiops* has a hamular lobe – within the hollowed hamulus of the pterygoid bone – and an anterior lobe. These two lobes are formed by an indentation of the pterygoid sinus, caused by the lateral laminae of the pterygoid bone and palatine bones. This bony structure is lacking in the pygmy sperm whale (*Kogia breviceps*) and thus, distinction of those lobes is meaningless. At its caudal end, the Pty sinus connects to three smaller sinuses, all of which are relatively close to the region occupied by the ear, the TMJ, and the attachment of the hyoid apparatus via the tympanohyal cartilage: the most lateral of these three sinuses (the middle sinus) is associated with the TMJ; a slightly more caudomedial sinus (posterior sinus) is associated with the tympanohyal joint and is bordered by the paroccipital crest; and a caudomedial sinus (peribullar sinus) that helps separate the tympanic bulla from the adjacent bones.

### Bony walls

Despite the variable geometry of the recesses and fossae of the sinus system, the bony associations of the sinuses follow some similar patterns among all odontocetes studied thus far. Delphinid species show marked similarity among the different species, while kogiids, physeteriids, and ziphiids all show similarity between themselves. In delphinids, the lateral wall of the pterygoid sinus is partly encased by the bony lateral laminae of the pterygoid and palatine bones. Conversely, in deep diving species like ziphiids, kogiids, and physeteriids, the lateral wall of the Pty sinus is composed entirely of soft tissue capable of collapsing onto itself. When manually manipulated, the Pty sinus is easily closed under the weight of the surrounding tissues, suggesting that maintaining it in an expanded state may require a degree of pressurization. Although the significance of these features is unknown, it is difficult to ignore the common threads among the non-delphinid deep diving cetaceans. Shallow divers such as *Tursiops*, have a deep indentation in the Pty sinus. In contrast, deep divers such as *Kogia* lack or have less distinct anterior and hamular lobes.

### Bony recesses

The sinuses of the accessory sinus system follow the contours of slight depressions (fossae; Mead and Fordyce, 2009) in the skull bones on the ventral basicranium (Figure 2); these depressions typically have a smoother surface than the other regions of these skull bones. The relatively large Pty sinus extends from the palatine and maxillary bones rostral to the orbit and caudally to the region of the Tym of the middle ear. The Tym sits in a deep recess bordered medially by the basioccipital bone. The pharyngeal crest (Mead and Fordyce, 2009), a ridge of bone that delineates the bony lateral margins of the pharynx, is formed by the ventrally projecting crests of the Pty and Boc bones. Note how the lobes of the pterygoid sinus in *Tursiops* extend much farther into the orbit than those of *Kogia*; these lobes join distally to form the supraorbital lobe of the Pty sinus, on the ventral aspect of the supraorbital crest of the frontal bone.

### Eustachian tube

The ET connects one half of the accessory sinus system and the ipsilateral middle ear to the respiratory system (Figure 2). The ET is a distinct, soft tissue, mucosa-lined, hollow tube located mostly within the lumen of the Pty sinus, attached to its ventral mucosa. The dorsal opening of the ET is within the essentially vertical nasal cavity just above the palatopharyngeal muscle, which acts as a sphincter to isolate the nasal cavity from the rest of the upper respiratory system during a dive. The ET extends ventrally within the nasal cavity to the Eustachian notch (Mead and Fordyce, 2009; pterygoid notch – Fraser and Purves, 1960; tubal notch – Schulte, 1917), which is a distinct cleft in the pterygoid bone on the ventral aspect of the skull (Figure 2). Near the apex of the Eustacean notch, the ET enters the accessory air sac system by passing through the wall of the Pty sac and a layer of connective tissue (which spans the Eustacean notch) to enter the internal bony nares of the respiratory system. The ET enters (but does not open to) the accessory sac system at the lateral aspect of the Eustachian notch. Within the Pty sinus the ET extends caudally, roughly parallel to the pharyngeal crest, to open just rostral to the opening of the middle ear cavity. In *Tursiops*, we have observed that the ET is surrounded by part of the adjacent pterygoid and peribullar venous plexus, however the luminal surface of the ET is also trabecular but has never injected with casting latex, suggesting it is a non-vascular structure. The caudal end of the ET is open to the lumen of the Pty sac rostral to the bony opening of the Tym – proximally in *Tursiops*, distally in *Kogia*.

### Blood-filled part (of the accessory sinus system)

All of the aforementioned lobes of the accessory sinus system are, in some form, associated with a venous plexus. The Pty sinus proper is associated with the most extensive venous plexus; however, all of the lobes are surrounded by or associated with a sizeable venous plexuses. Interestingly, the portions of the accessory sinus system along the basicranium are associated with venous plexuses directly connected via sizable emissary veins to the intracranial veins draining the brain.

### Fat bodies

**Figure 6:**
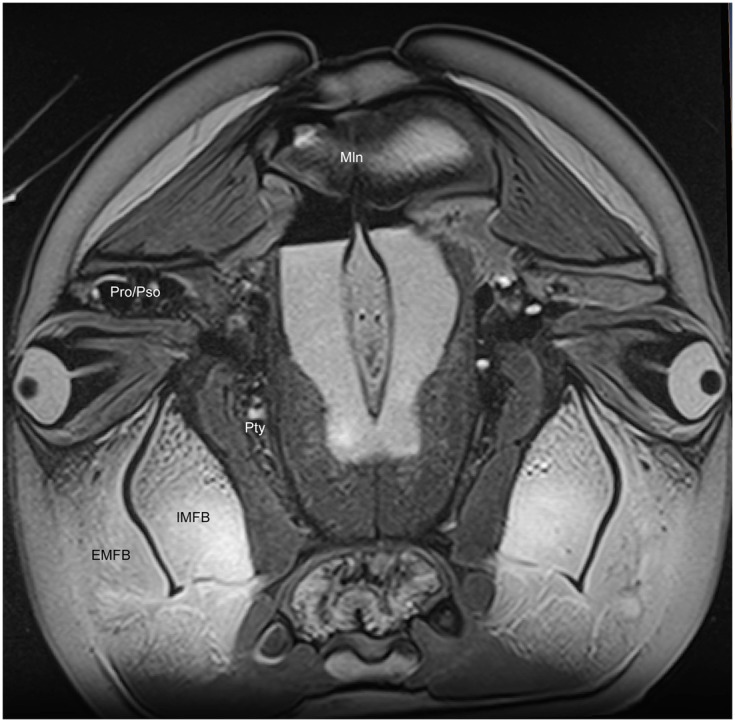
**Magnetic resonance imaging cross-sectional view of the head of a bottlenose dolphin at the level of the eyes, showing association of the intra- (IMFB) and extramandibular fat bodies (EMFB)**. Also labeled are portions of the melon (Mln), pterygoid sinus (Pty) and connection between the preorbital (Pro) and postorbital (Pso) lobes of the pterygoid sinus system.

**Figure 7:**
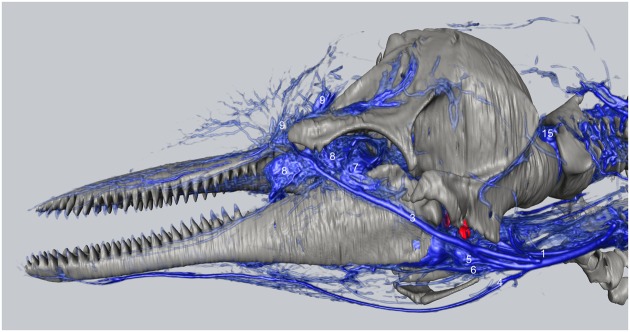
**Three-dimensional angiographic reconstruction of the left lateral aspect of the head of the bottlenose dolphin, showing associations of superficial veins and bony elements**. Veins were assigned names as follows: (1) internal jugular, (3) facial vein, (4) submental vein, (5) mandibular vein, (6) maxillary vein, (7) external ophthalmic plexus, (8) anterior lobe plexus, (9) melon veins. The red structure represents the tympanoperiotic complex. The external jugular is located medial to the facial vein and can therefore not be seen.

**Figure 8:**
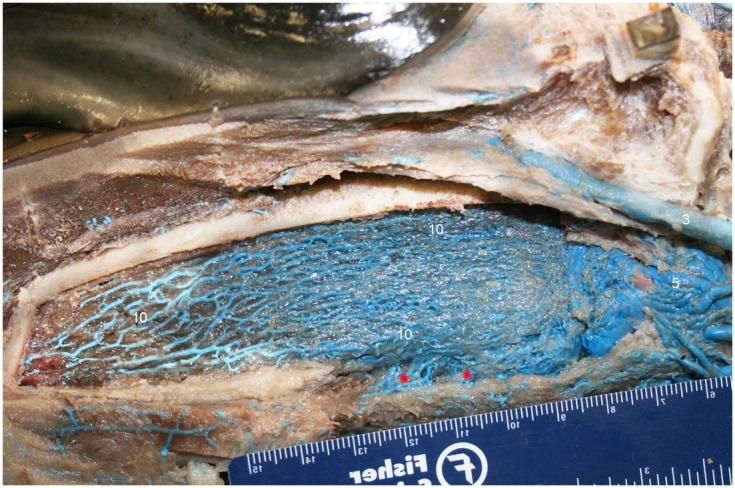
**Lateral view of right dentary (image has been flipped to simulate left lateral orientation for consistency between images) with a window cut out of the lateral wall in order to visualize the intramandibular fat body plexus**. Veins were assigned names as follows: (3) facial, (5) mandibular, (10) intramandibular fat body plexus. Note that red asterisks show the main connection between the intramandibular and extramandibular fat body veins.

**Figure 9:**
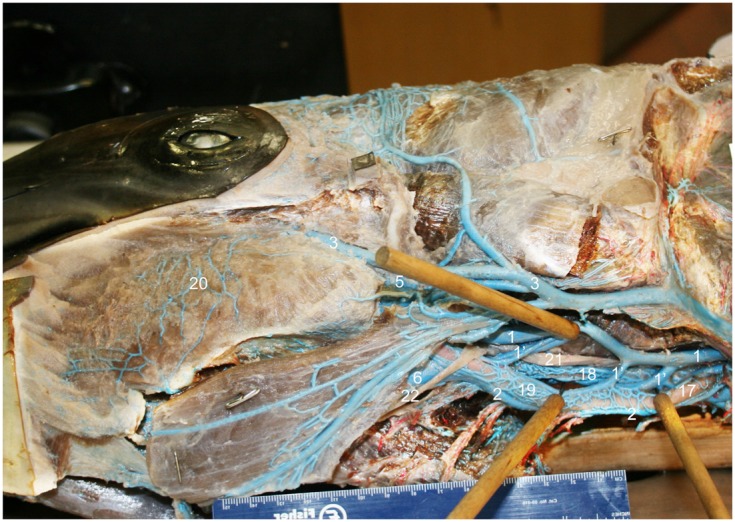
**Ventrolateral view of the right side of the bottlenose dolphin neck showing the jugular and facial veins, and the complex anastomoses between the structures (image has been flipped to simulate left lateral orientation for consistency between images)**. Structures were assigned names as follows: (1) internal jugular, (1′) anastomotic branches to the external jugular and to plexus surrounding external carotid artery, (2) external jugular, (3) facial, (5) mandibular, (6) maxillary, (17) external carotid artery, (18) internal carotid artery with surrounding venous plexus, (19) venous plexus surrounding the external carotid artery (*vena plexi commitans arteria carotidis externa*), (20) extramandibular fat body plexus, (21) vagus nerve, (22) hypoglossal nerve.

**Figure 10:**
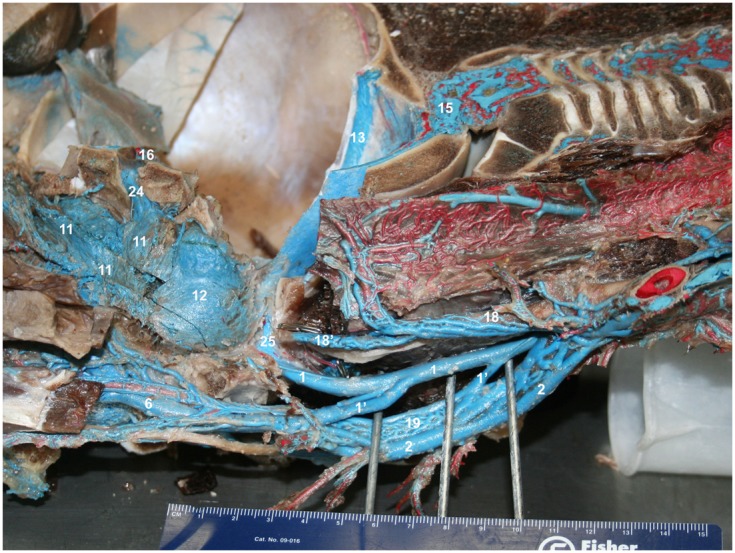
**Ventromedial view of the right half of a mid-sagittally sectioned bottlenose dolphin showing the jugular branching patterns**. Veins were injected with blue latex and arteries with red. Structures were assigned names as follows: (1) internal jugular, (1′) anastomotic branches to the external jugular and to plexus surrounding external carotid artery, (2) external jugular, (6) maxillary, (11) pterygoid plexus, (12) peribullar plexus, (13) temporal sinus, (15) epidural veins, (16) cavernous sinus, (18) internal carotid artery with surrounding venous plexus, (18′) regressed terminus of internal carotid artery, (19) external carotid artery with surrounding venous plexus, (24) emissary vein of foramen ovale, (25) emissary vein of jugular foramen.

We have observed extensive vascularization in three of the largest acoustic fat bodies of dolphins, namely the melon, intra-mandibular fat body (IMFB), and extra-mandibular fat body (EMFB) (Figures 1 and 6–8) in agreement with the observations of Fraser and Purves (1960), Maxia et al. (2007), and Slijper (1936) (for more complete descriptions of the fat bodies see Norris, 1969; Norris and Harvey, 1974; Cranford et al., 1996, 2008a; Koopman et al., 2003; Scano et al., 2005; Harper et al., 2008; McKenna et al., 2011). The melon fat is located on the dorsorostral aspect of the skull, extending from the rostral border of the nasal passage to the apex of the melon. Ventrally, the melon fat is separated from the ventral structures of the head by the vomer, maxillary, premaxillary, pterygoid, and palatine bones and the mesorostral cartilage. Each IMFB is located along the medial margin of the ipsilateral dentary bone. The dentary is hollow medially, along the caudal two-thirds of its length, and this cavity is filled with the IMFB which extends caudally to attach to the tympanoperiotic complex by way of dorsal and ventral branches of the fat body (Norris and Harvey, 1974; Ridgway, 1999; Cranford et al., 2008a). Medially, the lateral pterygoid muscles flank the IMFB, while its lateral border is defined by the dentary (Fraser and Purves, 1960). Each EMFB borders the lateral surface of each dentary. Along their ventral aspects, which extend ventral to the dentary, the EMFB and IMFB merge without any grossly visible distinction. On its lateral aspect the EMFB integrates seamlessly with the blubber surrounding the lower jaw. Although the individual veins draining each of these fat bodies are different, all three drainage fields eventually converge into the external jugular (#2, Figures 7–10) and facial veins (#3, Figures 7–9) and via anastomoses (#1′, Figures 9 and 10) into the internal jugular veins.

The melon fat is primarily drained by a multitude of veins that converge with the veins draining the rest of the tissues of the region (#9, Figures 7, 13 and 14) such as the nasal apparatus, maxillae, and maxillary (dorsal) lips. These veins converge, somewhat, as they travel through the dorsal infraorbital foramina of the maxillary and premaxillary bones, emerging on the ventral aspect of the skull as part of the plexus of the accessory sinus system and determined herein to be the dorsal continuation of the maxillary vein component of the pterygoid venous plexus (#6′′′, Figures 14 and 15E). From there, the plexus receives input from the nasal (#6′′), palatine veins (#6′′), cavernous sinus (#16), ventral petrosal sinus (#27), intramandibular plexus (#10), pterygoid venous plexus (#11), before coalescing into a single maxillary vein (#6) that follows the pterygoid crest to become the external jugular vein (Figures 7, 12 and 13). Additional but less voluminous drainage of the melon also likely occurs through various terminal branches of the facial vein that wraps around the antorbital notch (#3, Figures 7 and 9).

**Figure 11:**
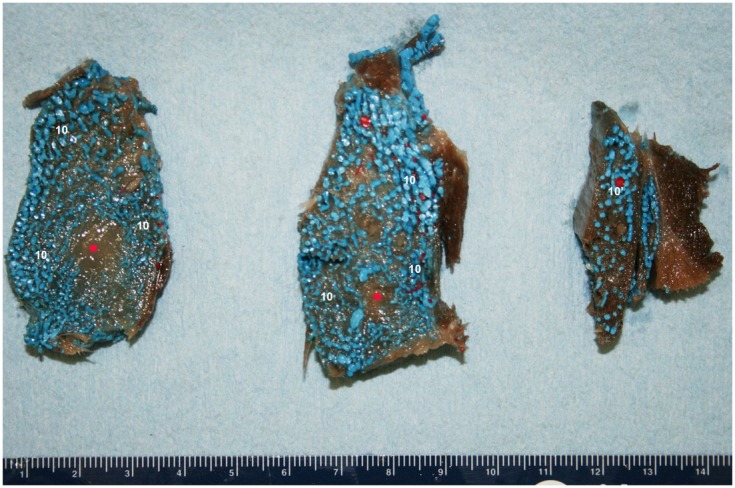
**Cross-sectional view of the intramandibular fat body (IMFB) of a bottlenose dolphin, progressing from caudal (left) to rostral (right)**. Note the extensive investment of the fat with a venous plexus (10). Also note the peri-arterial venous rete (10′-PAVR) surrounding the red mandibular alveolar artery in the dorsal region of the fat body, and the regions of un-injected fat highlighted with red asterisks near the ventral aspect. Due to the location and association with the concomitant artery, the PAVR has been termed the mandibular alveolar plexus.

The IMFB is invested with an intricate, voluminous plexus of anastomosing small caliber veins (#10, Figure 8). On cross section, the plexus can be seen throughout virtually the entire substance of the IMFB (#10, Figure 11), except for a small roughly circular region near its ventral margin (red asterisks, Figure 11). Histological quantification of vessel density has not yet been performed, however numerous specimens with latex-injected vasculature have consistently shown this well-defined region of reduced vascularization. Throughout its length, this un-vascularized region of fat forms a circular tube extending along much of the length of the IMFB. Interestingly, its position changes caudally as the tube approaches the ear, where it bends dorsad and attaches directly on the tympanoperiotic complex. Although the venous plexus appears undifferentiated in the way it invests the IMFB, the dorsal aspect of the plexus appears to have an increased density of longitudinally oriented veins that extend rostrally. A portion of the increased dorsal venous density contributes to the formation of a peri-arterial venous rete (PAVR), a rosette of veins that surrounds and follows the mandibular alveolar artery rostrad and has therefore been termed the mandibular alveolar plexus (#10′, Figures 11 and 13). The venous investment of the EMFB (#20, Figure 9) appears more diffuse and the drainage field less singular than that of the IMFB. Unlike the IMFB, the venous investment of the EMFB does not appear as dense, however despite its more diffuse nature, the veins of the EMFB still maintain a plexiform arrangement of notable extent.

### Vascular anatomy

**Figure 12:**
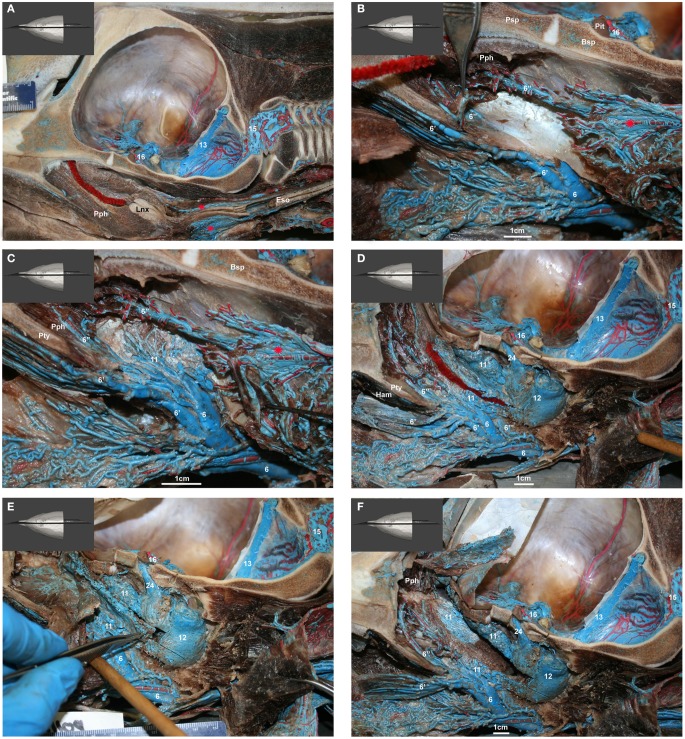
**(A–F)** Medial view of gross dissection of a bottlenose dolphin with latex-injected vessels. Approximate scale bars have been added whenever a ruler was not present in the photograph. **(A)** Shows the location of the pharyngeal plexus (red asterisks) in relation to the esophagus (Eso) and laryngeal cartilages (Lnx). The red structure represents the nasal passage surrounded by the palatopharyngeal muscle (Pph). **(B)** Shows the pharyngeal plexus (red asterisk), the maxillary vein (6) sending a palatine plexus (6′) that invests the roof of the oral cavity, and contributing to the nasopharyngeal veins (6′′) that drain the palatopharyngeal muscles (Pph). Also visible are the cavernous sinus (16), pituitary gland (Pit), and presphenoid (Psp) and basisphenoid (Bsp) bones that form part of the floor of the brain case. **(C)** Shows the contribution of the maxillary vein (6) to the formation of the lateral wall of the pterygoid plexus (11). Also visible is the pharyngeal plexus (red asterisk). **(D)** Shows the medial aspect of the peribullar plexus (12) and dorsal pterygoid plexus (11) with the bone of the pterygoid crest removed. Also visible are the emissary vein (24) traveling through the oval foramen, the temporal sinus (13) and epidural venous plexus (15). The red structure represents the Eustachian tube which extends from the tympanic bulla to the pharynx along the pharyngeal crest. Note the visible blue latex within the hamular (Ham) lobe of the Pty. This portion of the plexus can displace the entire volume of air within the hamular lobe. **(E)** Shows the dissection of the dorsal pterygoid and peribullar plexuses. **(F)** Shows the opened pterygoid and peribullar sinus, exposing part of the internal sinus lining and the connection between the lateral portion of the pterygoid venous plexus (11) that connects to the IMFB plexus. Note that the small inset panels within each larger panel show the orientation of the plane of section for each panel.

**Figure 13:**
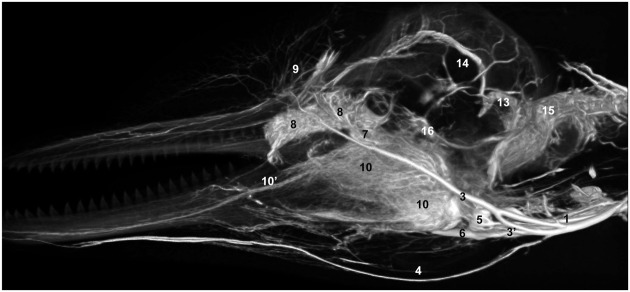
**Lateral view of a volume rendering of computed tomographical angiography of a bottlenose dolphin head showing complexity of venous investment**. The bone has been removed in the rendering to allow viewing of the spatial relationships between the superficial and deep venous structures. Veins were assigned names as follows: (1) internal jugular, (3) facial, (3′′) anastomotic branch to the maxillary vein, (4) submental, (5) mandibular, (6) maxillary, (7) external ophthalmic plexus, (8) anterior lobe plexus, (9) melon veins, (10) intramandibular fat body plexus, (10′) mandibular alveolar plexus, (13) temporal sinus, (14) dorsal sagittal sinus, (15) epidural venous plexus, (16) cavernous sinus.

**Figure 14:**
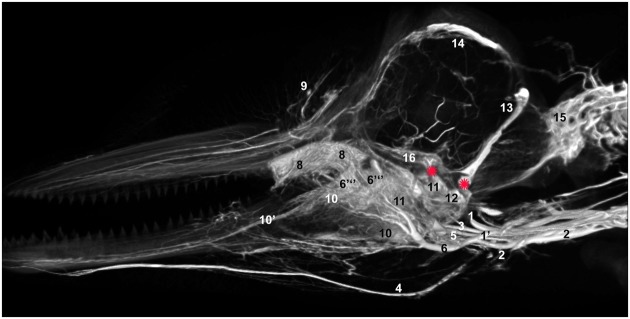
**Mid-sagittal view of a volume rendering of computed tomographical angiography of a bottlenose dolphin head showing complexity of venous investment along medial aspect**. The bone has been removed from the rendering to allow viewing of all venous structures. Veins were assigned names as follows: (1) internal jugular, (1′) internal jugular anastomosis with external jugular, (2) external jugular, (3) facial, (4) submental, (5) mandibular, (6) maxillary, (6′′′) dorsal continuation of the maxillary vein, (8) anterior lobe plexus, (9) melon veins, (10) intramandibular fat body plexus, (11) pterygoid sinus plexus, (12) peribullar sinus plexus, (13) temporal sinus, (14) dorsal sagittal sinus, (15) epidural venous plexus, (16) cavernous sinus. Note the two red asterisks showing the two basicranial emissary connections between intracranial and extracranial veins. These emissaries form robust connections between the plexuses of the pterygoid sinus system and the dural sinus system.

**Figure 15:**
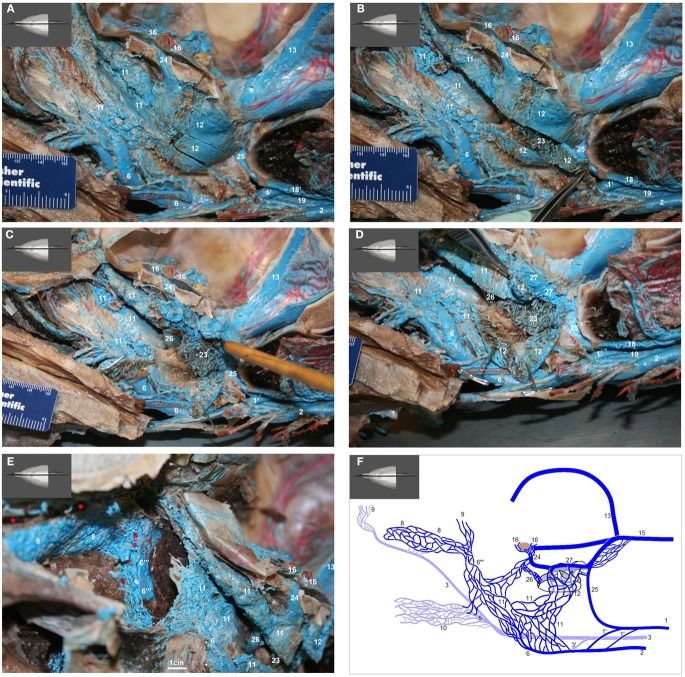
**(A–E)** show medial view of gross dissection of the pterygoid and basicranial regions, identifying some of the key venous structures outlined in the text (structure numbers correspond to those in previous images). **(A)** Shows the medial wall of the plexuses associated with the accessory sinus system and their emissary connections (24 and 25) to the intracranial dural sinuses. **(B)** Shows the pterygoid (11) and peribullar (12) plexuses being opened, to see the lateral wall of the pterygoid sinus plexus and the *corpus cavernosum* of Boenninghaus (23). **(C)** is similar to **(B)** but with the peribullar sinus opened further to expose the *bulbous* *venosus epibularis* of Boenninghaus (26) emerging from within the tympanic bulla (red asterisk) and merging with the dorsal part of the pterygoid and peribullar plexuses. **(D)** is similar to **(C)** but with the dorsal aspect of the peribullar plexus opened further to expose the ventral petrosal sinus (27) and its caudal connection to the internal jugular emissary vein of the jugular foramen (25). The tympanic bulla is marked with a red asterisk. **(E)** Shows an oblique rostral view of the left dorsolateral extension of the pterygoid plexus (11) around the external pterygoid muscle (Pte) as it forms the dorsal continuation of the maxillary vein (6′′′) that passes through the infraorbital foramina. Also shown is the extensive and complex nature of the anterior lobe plexus (8) and the anterior lobe (red asterisks) it is associated with. **(F)** Shows a simplified schematic illustration of the medial view based on a modification of Boenninghaus’ (1904) illustration of the venous connections in the pterygoid and basicranial regions. Shown are the main patterns of venous connections of the plexuses associated with the intramandibular fat body, accessory sinus system and the intracranial dural system. Lighter blue vessels are more lateral (behind) darker structures. The brown structure represents the pituitary gland. Note that the *corpus cavernosum* of Boenninghaus (23) is not illustrated here due to its location lateral (behind) the peribullar plexus (12).

As is common to most domestic mammals (e.g., cow, horse, pig, cat), the external jugular vein of the bottlenose dolphin is the main drainage route of the pterygoid vasculature although the internal jugular vein can be the primary drainage (e.g., dog; (Ghoshal et al., 1981; Nickel et al., 1981; Evans, 1993; Schaller, 2007). What is different in the dolphin, however, is that the linguofacial vein branches off of the brachiocephalic trunk instead of the external jugular vein, and in some cases as a common branch with the internal jugular vein. Additionally, the facial vein gives off the mandibular vein that in domestic mammals originates from the maxillary vein – the main continuation of the external jugular vein – or one if its tributaries. As we are just now beginning to elucidate the vascular anatomy of deep diving cetacean species, the following description of venous branching patterns is based solely on that observed in *Tursiops*. Additional clarification can be obtained in the (Movie S1 Supplementary Material) provided. Table 2 shows the structures labeled in Figures 7–15 and their corresponding names. The plexuses associated with the mandibular fat bodies and accessory sinus system are drained primarily by three parent veins, namely the facial, external jugular, and internal jugular veins.

**Table 2:** **List of soft tissue (blood vessels and nerves) structure labels used in the figures and their corresponding names**.

Structure label	Structure name
1	Internal jugular vein
1′	Anastomotic branch to external jugular vein
2	External jugular vein
3	Facial vein
4	Submental vein
5	Mandibular vein
6	Maxillary vein
6′	Palatine plexus
6″	Nasopharyngeal veins
6′′′	Dorsal maxillary vein continuation
7	External ophthalmic plexus
8	Anterior lobe plexus
9	Melon veins
10	Intramandibular venous plexus
10′	Mandibular peri-arterial venous rete/mandibular alveolar veins
11	Pterygoid plexus
12	Peribullar plexus
13	Temporal sinus
14	Dorsal sagittal sinus
15	Epidural veins
16	Cavernous sinus
17	External carotid artery
18	Internal carotid artery
18′	Regressed terminus of internal carotid artery
19	Venous plexus surrounding external carotid artery
20	Extramandibular venous plexus
21	Vagus nerve
22	Hypoglossal nerve
23	*Corpus cavernosum* of Boenninghaus
24	Emissary vein of oval foramen
25	Emissary vein of jugular foramen
26	*Bulbous venosus epibularis* of Boenninghaus
27	Ventral petrosal sinus

### Facial vein

At about the level of the paroccipital crest and just lateral to the skull attachment of the tympanohyal element, the facial vein trifurcates into large lateral and ventromedial veins and a midline coalescing plexiform mass of small veins^2^ (#3, 5 and 6, Figures 7–9 and 13).

(1) The lateral branch of the facial vein (#3, Figures 7–9 and 13) extends dorsolaterally around the dentary, passing at an oblique angle under the mandibular condyle and crossing the condyloid crest on its path to the external ophthalmic plexus (#7) and the antorbital notch of the maxillary bone. In addition to its medial connection through the orbit to the external ophthalmic plexus, the vein continues around the antorbital notch to the dorsal aspect of maxillary and premaxillary bones to drain the tissues of the melon, nasal plugs, and dorsal lips (#9, Figures 7 and 13). This vein is consistent in location and drainage field to the facial vein of terrestrial mammals.
(2) The middle branch of the facial vein (#5, Figures 7–9 and 13) can arise as either a single branch or a brush-like spray of small veins that extend from the rostral margins of the of the facial vein trifurcation. Whether single or plexiform, the structure eventually becomes a spray of numerous small veins that enter the dense connective tissues associated with the ear and lower jaw attachment to the skull. Upon entering the connective tissue, the veins contribute – along with the pterygoid plexus described below–to the formation of the fibrovenous plexus described by Fraser and Purves. This fibrovenous plexus is composed of an intricate network of small (∼1–5 mm diameter) caliber anastomosing veins that invest much of the connective tissue and wrap around part of the tympanic bulla of the ear bone complex. The fibrovenous plexus^3^ extends rostrally, gradually losing its connective tissue component as it transitions into the soft fatty tissues of the IMFB. As the ventrolateral portion of the fibrovenous plexus extends into the IMFB to become the mandibular plexus, the veins appear to distribute throughout most of the fatty substance (#10, Figures 8, 11, 13 and 14). Along its dorsal margins the IMFB plexus coalesces to form a PAVR (#10′, Figures 11 and 13) that surrounds the mandibular artery and courses rostrad toward the mental foramina of the distal dentary and mandibular symphysis, occasionally giving off branches to the teeth. This PAVR is consistent with the mandibular (inferior) alveolar vein described in terrestrial mammals and is therefore responsible for draining blood from the teeth and rostral portions of the lips of the lower jaw.
(3) The third and most medial branch of the facial vein (#6, Figures 9, 10, 14 and 15) is similar in size to the lateral branch. Approximately 5 cm after the ventromedial branch emerges from the trifurcation it is joined by the maxillary vein, the main terminus of the large external jugular vein (#2, Figures 9, 10, 14 and 15). This medial branch of the facial vein might, therefore, be considered an anastomotic branch to the maxillary vein. The two veins join to form a larger vein that travels roughly horizontally to the level of the ceratohyal cartilages of the hyoid apparatus. At that level, the vein gives off smaller lingual and pharyngeal branches before curving dorsad along the pterygoid crest. It should be noted that the lingual vein – usually arising from the maxillary vein further rostrad – can occasionally branch off of the facial vein as a common trunk with the aforementioned ventromedial anastomotic branch to the maxillary vein.

### Internal jugular vein

The internal jugular vein (#1, Figures 7, 9, 10, 13 and 14) can arise either singularly or as a common trunk with the linguofacial vein, directly from the brachiocephalic vein. Once the internal jugular vein has traversed the short neck and reached the head, it contributes to the drainage of three regions.

(1) The first and most proximal (caudal) contribution is formed by numerous anastomoses (#1′, Figures 9, 10, 14 and 15) between the internal and external jugular veins. These anastomoses arise from the ventral aspect of the internal jugular vein and travel obliquely ventrorostrad to fuse with the external jugular plexus surrounding the external carotid artery (#19, Figures 9 and 10), in agreement with the findings of Ridgway et al. (1974). Like the mandibular alveolar vein, the plexus surrounding the external jugular vein forms a PAVR. From this point rostrad, the external jugular vein (#2) becomes the maxillary vein (#6) and drains the majority of the pterygoid plexus as well as parts of the nasopharyngeal, palatine, and dorsal nasal regions. Due to the anastomoses, the internal jugular vein might therefore be considered to facilitate this drainage.
(2) The second and third branches arise in common from a bifurcation of the distal internal jugular vein. The proximal of the two branches curves sharply rostrad to become the ventral petrosal sinus (#27, Figure 15D) that lines the dorsal aspect of the peribullar plexus (#12, Figures 14 and 15).
(3) The third branch extends vertically from the bifurcation of the internal jugular vein, to enter the jugular foramen as the terminus of the internal jugular vein (#25, Figures 15A–D). As it enters the caudal portion of the calvarium, it fuses with the temporal/sigmoid/transverse dural sinus, to form one of the main drainage paths for blood from the brain case.

### External jugular vein (#2, figures 9, 10, 14 and 15)

The external jugular vein arises directly from the brachiocephalic trunk as a plexiform structure composed of small and large veins that surround the external carotid artery. The ventral portion of this plexus is highlighted by a distinct vein of considerably larger caliber – the external jugular vein – than the rest of the plexus veins. As the external jugular vein traverses the neck and approaches the head, it receives the anastomotic branches (#1′, Figures 14 and 15) of the internal jugular (#1) and facial veins (#3). It finally receives an anastomotic branch from the facial vein and then curves medially to follow the basioccipital and pterygoid crests as the maxillary vein (#6). Along its course through the pterygoid region, the external jugular vein forms numerous branches and countless small anastomoses with other venous tributaries. The main branches invest the palatine, nasopharyngeal, pterygoid, and dorsal head tissues as follows:

(1) On its course to the rostroventral aspect of the brain case, the maxillary vein sends dorsorostrad oriented veins of a plexiform nature (#6′, Figures 12B–D) that invest the tissues on the roof of the oral cavity. These veins were considered to be the palatine veins which are shown to form a palatine plexus.
(2) Before it breaks up into the countless veins that compose the pterygoid plexus (#11, Figures 12C–E, 14 and 15) that invests the lining of the pterygoid sinus and drain the substance of the pterygoid muscles, the maxillary vein sends rostrodorsad oriented branches (#6′′, Figures 12B–F) into the palatopharyngeal muscles where they anastomose with other palatopharyngeal veins supplied by the external jugular vein and a large pharyngeal plexus that surrounds the rostral esophagus and laryngeal cartilages (red asterisks, Figure 12A). At this level the maxillary vein loses its singular identity as it breaks up to become part of the pterygoid plexus associated with the accessory sinus system (#11, Figures 12C–F, 14 and 15). Nonetheless, the lateral region of the plexus extends laterally under the external pterygoid muscle – such that is forms a cradle for the muscle – and fuses with dorsal extensions of the intramandibular plexus. The combined plexuses extend dorsad toward the maxillary bone and narrow considerably as they approach the ventral aspect of the maxillary and premaxillary bones. The plexus surrounds all but the medial aspect of the maxillary artery as both structures travel to the infraorbital foramina (#6′′′, Figures 14 and 15E). This dorsal extension of the venous plexus maintains its course to the ventral infraorbital foramina, in juxtaposition with the corresponding maxillary artery, and is therefore considered herein as the terminus of the maxillary vein. Just before the plexus reaches the infraorbital foramina, its rostral aspect sends a large mass of highly convoluted veins rostrad. These veins form a robust venous plexus (preorbital and anterior lobe plexus) that travels along the ventral lining of the anterior lobe of the accessory sinus system (#8, Figures 7 and 13–15).

## Discussion

### Significance of the anatomy

Despite the significant reorganization of the skull bones to accommodate dorsally located nares and a large melon, many of the major veins in the heads of odontocetes find analogs in domestic mammals. Similarly, although we have no developmental data on these venous structures, the striking similarity in distribution and location of many of those structures suggests that they are also homologs of the veins seen in domestic mammals. Nonetheless, a few significant elaborations on structures were observed, especially in regions associated with acoustic fat bodies and air sinuses. Specifically, given the novel evolutionary nature of the cetacean mandibular fat bodies, the venous plexuses of the intra- and EMFBs find no notable homolog in domestic mammals. Although it is unknown whether or not the venous plexuses of the accessory sinus system might find homologs in the pterygoid plexuses of various domestic mammals such as horses, cows, and dogs, the complexity and volume of the plexus system of cetaceans appears unparalleled.

### Accessory sinus system

Since the accessory sinus system is not completely encased in robust, rigid bony compartments, it is presumably exposed to the effects of changing barometric pressures encountered during a dive. In order to avoid pathologies associated with physical injury of tissues exposed to dysbaric changes it seems safe to assume that there exists a significant amount of physiological flexibility within those structures. The apparent susceptibility of the accessory sinus system to external pressures may have significant implications on diving gas kinetics. The intimate anatomical association between large venous masses with thin linings and pockets of air exposed to changing pressures begs a question regarding absorption and clearance of gases during a dive (Reidenberg and Laitman, 2008). With increasing hydrostatic pressures, gases in air are progressively driven into solution. This phenomenon is responsible for increased absorption of nitrogen in the lungs of a scuba diver. Marine mammals had long been thought to be exempt from significant accumulation of nitrogen during dives because they do not breathe compressed air at depth, and because their pulmonary alveoli are believed to progressively collapse with depth, thereby isolating pulmonary gases away from absorptive respiratory surfaces (Scholander, 1940). Since the pressurized pulmonary air is thought to be segregated from the pulmonary blood circulation, nitrogen absorption is believed to be limited (Kooyman, 1973; Ridgway and Howard, 1979). Yet these facts seem contradictory to the findings of gas emboli in beaked whales, as diving-related gas emboli in humans are typically associated with pulmonary injury or autochthonous bubble growth due to improper management of nitrogen gases and/or air absorbed at depth (Brubakk et al., 1999; Neuman, 1999). Indeed, recent studies have begun to show that gas emboli within stranded deep diving cetaceans are composed primarily of nitrogen gas, adding further support to the notion that under certain circumstances, nitrogen saturation may be possible (Bernaldo de Quirós et al., 2011, 2012). Additionally, recent gas modeling in cetaceans has shown that the risk for nitrogen saturation may in fact be a concern (Houser et al., 2001; Fahlman et al., 2006, 2009; Hooker et al., 2009, 2011).

Fraser and Purves (1960) stated that the elaborate plexus investing the air sac system was “apparently entirely subservient to the proper functioning of the latter.” Given the intimate association of the accessory sinus system with the aforementioned venous plexuses, this seems like a reasonable conclusion. We suggest that the large venous investment of the air sinuses may provide an alternate mode of nitrogen absorption or elimination during the course of a dive. Such gas kinetics may be beneficial during normal diving, but may pose a threat of DCS or venous gas embolism when diving conditions are artificially or inappropriately altered. During descent, the increasing pressure could drive nitrogen out of the sinus and into the surrounding venous plexus, as happens in the lungs. Admittedly, whether such diffusion across the venous wall is possible will likely depend on the thickness of the venous wall and the nitrogen partial pressure differential between the venous blood and sinus air.

Although Fraser and Purves (1960) conducted some histological examinations of the lining of these sinuses, we suggest that future studies conduct histomorphometry in order to facilitate evaluation of the likelihood of gas exchange across those surfaces. Since the venous plexuses are not composed of capillary beds but rather veins of variable size, diffusion across their walls at normal pressures is likely to be minimal or non-existent, but would instead require considerable pressure differentials to drive gas across. Modeling of such a function may provide insights in the absence of physiological data. Although a reasonable assumption may be that only as much nitrogen as is present in the sinus could ultimately be absorbed, the intranarial connection between the pulmonary and accessory sinus system should not be ignored, since decreasing gas volume in the sinuses could be supplemented by pulmonary air in order to enable equalization of the middle ears. Interestingly, if indeed nitrogen gas can be exchanged at the lining of the accessory sinuses, a reduction in pressure – as happens during ascent – could result in reversal of nitrogen flow from the blood to the sinuses, providing a non-pulmonary mechanism for elimination of nitrogen from the blood. During ascent, reducing pressure in the sinuses may be able to draw nitrogen out of the venous blood lining the sinuses. Nitrogen could then be cleared through the ET and out of the nasal passages. A recent analysis of the gas found in the pterygoid sinuses of stranded cetaceans showed that the sinuses have consistently high nitrogen gas levels (Bernaldo de Quirós et al., 2012). Additionally, elevated levels of CO_2_ were also found, suggesting that diffusion of CO_2_ across the sinus membranes should also be examined. Given the complexities of working with and interpreting results from post-mortem specimens, more research on this topic is needed, however it may provide support for our suggestions.

Another possible function of the accessory sinus vasculature that was postulated by Fraser and Purves (1960) may involve the redistribution of blood into the venous plexuses to accommodate the reduction of air volume during descent. As suggested by Fraser and Purves (1960), this could allow the lost volume of air to be replaced with blood to avoid dysbaric trauma to the sinus tissues as well as facilitating hydrostatic equilibrium of the ears which are surrounded by the sinus system. Such a pressure-related redistribution of blood to the sinus plexuses may help explain the common presence of foam in the accessory sinus system, conceivably created by venous transudate that could help generate and stabilize the foam. Although such a blood-redistribution function may seem plausible in delphinids that have rigid bony lateral pterygoid laminae (Figure 2), it may not explain the presence of an intricate plexus in deep diving odontocetes that have a flexible lateral wall that can likely deform medially to accommodate the reduction in air volume consequent to compression. There is little doubt that deep diving marine mammals have evolved mechanisms for limiting nitrogen gas absorption and mitigating or managing bubble formation, whether *in situ* or intravascular. Nonetheless, given the extreme nature of the diving life styles of some of the deep diving cetaceans, it is possible that they live at the limits of physiological tolerance, with small margins for error regarding gas management. Retention of these vascular plexuses despite the modified accessory sinus system anatomy may reflect their need for greater control over gas management.

### Fat bodies

The acoustic fat bodies of the lower jaw are interesting on many levels and have been a topic of considerable research and debate. At the forefront is their presumed function as analogs to the external pinnae of other mammals, receiving and channeling sound to the ears (Norris, 1969; Norris and Harvey, 1974; Cranford et al., 1996, 2008a,b). This suggested function naturally implicates these structures in any discussion concerning auditory impairment resulting from intense ensonification. Perhaps less obvious is the possible role of the acoustic jaw fats as a source of fat emboli or as a nitrogen sink (Kooyman, 1973; Jepson et al., 2005). Koopman (2007) suggested that deep diving odontocetes such as beaked whales and sperm whales may fill their blubber with lipids that provide some type of physiological or mechanical advantage to diving (e.g., nitrogen sink). Mammalian fats have traditionally been considered poorly vascularized structures, and given the general paucity of cetacean vascular information, this paradigm has understandably been propagated in the field of cetacean biology and discussions of vascular investment of fats (Houser et al., 2001; Fahlman et al., 2006, 2009; Hooker et al., 2009). In contrast, our recent vascular research has shown that some cetacean fatty tissues are very well vascularized. As noted by Fraser and Purves (1960), extra- and intra-mandibular jaw fats are proving to be extensively vascularized by veins (see Figures 8, 9 and 11) and often well vascularized by arteries. Jepson et al. (2005) alluded to the possibility that damage to the tissue barrier between the acoustic lipid and the venous lumen could introduce fat into the circulation and lead to the formation of fat emboli. Such damage might result from physical trauma such as intense ensonification blunt or sharp force trauma, or from traumatic expansion of gas bubbles within nitrogen-saturated adipose tissue.

Two factors make the acoustic jaw fat interesting to us from this perspective. Firstly, fat is known to absorb nitrogen well and therefore can act as a sink for nitrogen that can expand once hydrostatic pressure is reduced (Lango et al., 1996). Secondly, the close anatomical association of these fat bodies and their venous plexuses to the gas-filled sinuses and extensive pterygoid vascular networks may place them at increased risk of receiving either (1) elevated nitrogen levels absorbed and accumulated through the sinus lining, or (2) gas emboli generated in the veins lining of the sinuses. Nitrogen absorbed through the sinus lining could travel to the fat bodies via the robust connections described, being absorbed by the fat body and expanding within the adipose tissue, or expanding within the IMFB veins and disrupting the vascular barrier between the blood and lipid. Any of the aforementioned situations could conceivably result in physical damage of the fat with subsequent release of lipid into circulation.

The high solubility of nitrogen in fat tissue is well-documented and results in high levels of nitrogen absorption in adipose tissue (Lango et al., 1996). Traditionally however it has been thought that nitrogen loading and unloading of fatty tissues is limited by the poor perfusion of the tissue (Fahlman et al., 2006, 2009). Since the acoustic jaw fats are indeed well vascularized, it is conceivable that they may exchange nitrogen at higher rates than are typically associated with fatty tissues. Interestingly, there appears to be an exceptionally disproportionate number of veins relative to arteries relative to most other tissues. Though no functional studies exist from which to draw any conclusions, we suggest that an elevated venous density would allow for faster nitrogen elimination and may provide a mechanism for rapid nitrogen clearance in a vital tissue that when damaged can significantly reduce an individual’s chances of survival. We also cannot ignore the fact that the large volume and high surface area construction of the IMFB venous plexus may facilitate regional heterothermy of the intramandibular fats that the veins invest. Altering the temperature of the fat could affect the degree of solubility of nitrogen and therefore not only affect nitrogen loading and unloading but also influence the ease with which lipid can be mobilized and introduced into the vasculature, resulting in fat embolization.

Another possibility is that by modulating the volume of venous blood in the mandibular acoustic fats, cetaceans may be able to alter the density of the fats either due to increased blood density or temperature change, thereby effecting a change in acoustic properties of the fat. This could allow cetaceans to either fine tune their hearing to specific frequencies or adjust the density of the fat to maintain their hearing in altered ambient temperature environments, as may happen during a transition from warm to cold water. Such functions may be supported by the finding that false killer whales are able to actively control their hearing based on the received echo from a target they are echolocating on (Nachtigall and Supin, 2008). Finally, we cannot ignore the possible effect that regional mandibular heterothermy may have on modulating other sensory components. A large portion of the inferior alveolar nerve of the mandibular branch of the trigeminal nerve passes through the intramandibular plexus on its course to the brain. If the intramandibular plexus is capable of producing temperature changes within the mandible, those changes may be able to modulate the sensory input from the nerve by affecting the amplitude, speed, and/or duration of the action potentials (Inman and Peruzzi, 1961; Ishiko and Loewenstein, 1961).

Numerous researchers have shown that temperatures of the blubber and extremities of cetaceans are often near ambient water temperatures, and since the mandible is a peripheral structure with high surface area it may frequently reach temperatures well-below normal mammalian core body temperature (Noren et al., 1999; Meagher et al., 2002, 2008; Barbieri et al., 2010). Temperatures below 20°C have been shown to cause sharp decreases in the amplitude of the action potential of rapidly adapting Pacinian corpuscles, while the action potential often disappears at temperatures below 15°C (Inman and Peruzzi, 1961; Ishiko and Loewenstein, 1961). Therefore, it may be possible for the mandibular plexuses to modulate the temperature of the surrounding tissues in order to overcome the adverse affects suboptimal temperatures may have on proper functioning of peripheral sensory nerves. Although this kind of temperature modulation of peripheral nerves might serve no apparent benefit in terrestrial mammals, the role of the inferior alveolar nerve is not understood in cetaceans and this implication should perhaps not be completely discounted given the conductive heat loss that occurs in water and the highly derived adaptations of cetaceans.

Interestingly, the melon fat appears to be drained by veins that are concentrated along the periphery of the fat body, with large veins avoiding a direct course through the main substance of the melon fat. Conversely, the IMFB is densely invested with veins. As we must assume that anatomical structures are not the result of evolutionary processes resulting in meaningless manifestations, we are compelled to believe that this distinct difference may be reflective of a functional role. It is possible that this reflects different functional needs to modulate physical characteristics of the fat bodies.

### Clarifications/inconsistencies

Interestingly, Fraser and Purves (1960) noted the presence of distinct pterygoid and maxillary veins, with the maxillary vein traveling lateral to the paroccipital process and tympanohyal, and merging with the IMFB plexus. They then noted that it merges with the deep temporal vein as it travels caudad. They made no mention of the maxillary vein’s proximal connection to the external jugular vein as is common in domestic mammals, and noted that the pterygoid vein eventually joined with the mandibular and internal jugular veins. They described the pterygoid vein as running along the pharyngeal crest for a considerable length, eventually terminating as countless ramifications of the fibrovenous plexus associated with the accessory sinus system. We found this description to be confusing for a number of reasons. First, in most domestic mammals (except dogs) the pterygoid vein is a branch of the maxillary vein, which like the linguofacial vein is one of the terminal branches of the external not internal jugular vein. Indeed, in our delphinid specimens the intramandibular plexus and subsequent mandibular alveolar veins were seen consistently arising as a branch of the facial vein. Secondly, our findings in *Tursiops* suggest that the structure described by Fraser and Purves (1960) as the pterygoid vein is in fact consistent in location and course with the maxillary vein – traditionally considered the terminal branch of the external jugular vein – and the pterygoid venous plexus is instead formed by numerous small pterygoid veins that branch off of the maxillary vein and by the subdivision of the maxillary vein itself. Thirdly, since the internal jugular vein enters the jugular foramen, the only large veins we have observed traveling lateral to the tympanohyal cartilage and proximal stylohyal bone are the continuation of the external jugular vein that wraps around the tympanohyal cartilage, and the facial vein (#3, Figure 7) which is far removed from this location as it courses just deep to the blubber layer and subcutaneous fat. Our findings are consistent with the patterns observed by Ommanney (1932) in fin whales (*Balaenoptera physalus*) and by Walmsley (1938) who noted that the maxillary vein of the fetal fin whale is formed by veins accompanying the external carotid artery and tributaries from the pterygoid venous plexus. Additionally, the vein described by Fraser and Purves (1960) as the pterygoid vein formed in our specimens the terminal branch of the external jugular vein and passed dorsad through the infraorbital foramina, a pattern consistent with that of the maxillary vein of domestic mammals. Finally, we were not able to identify any substantial veins in the location identified by Fraser and Purves (1960) as the maxillary vein, unless they were referring to one of the veins of the external ophthalmic plexus (#7; rete vena ophthalmica externa of Slijper, 1936) or one of the many sizable veins of the pterygoid venous plexus.

The aforementioned differences could be due to the fact that the specimens used by Fraser and Purves (1960) were decapitated specimens that may have been missing the more proximal branches of the jugular veins. Nonetheless, the illustrations presented by Fraser and Purves (1960) show the vascular and skull morphology from the occipital condyles rostrad, including the internal jugular vein, however the external jugular vein has been completely omitted, as has the origin of the maxillary vein. It may be that the proximal most trunk labeled as the internal jugular vein is in fact the common jugular, from which the internal and external jugular veins branch. The ventral branch (external jugular) would then give off the maxillary vein as its primary branch. It is also possible that due to the use of decapitated specimens, Fraser and Purves (1960) considered the anastomotic branch of the internal jugular vein – seen in all of our specimens – as the main drainage path of the mandibular and maxillary veins, since they neither mention nor illustrate the external jugular vein.

Another source of confusion for us was the reference made by McFarland et al. (1979) to the *rete vena ophthalmica externa* first noted by Slijper (1936). McFarland et al. (1979) showed a venous vascular cast of a large extracranial retial structure adjacent to the cranium and connecting to the spinal veins via the first three intervertebral foramina. McFarland et al. state that this rete lies in the lower jaw and is “probably the *rete vena ophthalmica externa*” described by Slijper (1936). In none of our specimens did the intramandibular plexus connect directly to the epidural veins. Although difficult to discern from the photographic perspective provided, the positional references seem inappropriate and we therefore respectfully suggest that the vascular cast may be of the plexus investing the parieto-frontal region that in our specimens had anastomoses with the superficial cervical and occipital veins and ultimately the epidural circulation. Our findings suggest that the rete pictured by McFarland et al. (1979) may in fact be composed of numerous structurally and regionally distinct retia that warrant separate nomenclature. Interestingly, despite the numerous fine caliber veins and plexuses that injected in our specimens, the only direct connections we observed between the spinal veins and the plexuses on ventral aspect of the skull were via the internal jugular emissary vein of the jugular foramen and the emissary vein of the foramen ovale. It is unclear at this time if this was an artifact of the injection medium, an individual variation, or a misidentification of structures; however it appears to be a significant difference. Given the degree of plasticity inherent in venous connections, it is possible that these connections manifest differently between individuals of a given species.

At the level of the medial aspect of the orbit, the lateral portion of the plexus associated with the Pty sinus fuses with dorsal offshoots of the intramandibular venous plexus and progresses dorsad to become the anterior lobe venous plexus. The largest portion of the anterior lobe venous plexus is located ventral and rostral to the eyestalk, and just ventral to the maxillary and premaxillary bones, in close association to the ventral lining of the preorbital and anterior lobes of the accessory sinus system. The anterior lobe venous plexus acts as a crossroads for numerous veins that converge on it. It has connections to intracranial blood via the ophthalmic plexuses that exit the orbital fissure, the melon via the facial vein and the infraorbital veins, and the ventral skull via the plexus of the accessory sinus system and maxillary vein. These collateral connections may be important for understanding compartmentalization of gases and observed distribution of gas and fat emboli.

Upon initial examination of the anatomy of the vasculature in the region of the Pty and peribullar sinuses, it becomes apparent that although there are homologies that may be drawn, the general pattern of venous investment seems relatively random. However, upon closer examination, it becomes evident that the locations and connections of the plexuses are relatively consistent between individuals, with only the localized branching patterns within the plexuses showing much variability. Nonetheless, it appears that in addition to those structures with likely homologies to generic mammalian structures, almost every available space between the jugular veins and distal head structures is filled with plexuses of anastomosing veins, forming what can only be considered a complex of countless collateral pathways. Although deciphering the exact anatomy of the venous structures themselves is important, the connections between those structures may be equally important from a functional perspective, as they form collateral pathways and alternate routes for transport of emboli and compartmentalization of gases.

The paired cavernous sinuses of domestic mammals form a ring-like venous structure around the pituitary gland (#16, Figures 12 and 15), just dorsal to the basisphenoid bone (Ghoshal et al., 1981). Rostrally the sinuses connect to the ophthalmic veins via the orbital fissure, while caudally they run confluent with the ventral petrosal and basilar sinuses. The ventral petrosal sinuses connect the caudolateral aspect of the cavernous sinuses to the ventral margin of the sigmoid sinuses (Evans, 1993). In *Tursiops*, these sinuses – located at the floor of the brain – connect the cerebral venous circulation to the plexuses of the accessory sinus system. Fraser and Purves (1960) noted that “the petrosal and cavernous sinuses of cetaceans are divested of the bony cranial protection found in terrestrial mammals, due to the displacement of the tympanoperiotic from participation in the wall of the cranium”. We found this statement confusing since the cavernous sinus of most domestic mammals lies in a similar position – relative to the basicranial bones – as what we observed in dolphins (Ghoshal et al., 1981; Evans, 1993; Schaller, 2007), namely on the dorsal aspect of the pre- and basisphenoid bones. Fraser and Purves (1960) then stated that in cetaceans the cavernous tissue body or spongy mass of Beauregard is the homolog of the cavernous sinus of terrestrial mammals. This too seems problematic since the spongy mass of Beauregard was illustrated by Boenninghaus (1904) as surrounding the tympanic bulla of the ear (if it is the same as the structure labeled *corpus cavernosum*), ventral to the ventral petrosal sinus. This is an unusual location for the cavernous sinus.

We suggest that the cavernous sinus of the bottlenose dolphin is in fact in the expected intracranial location just rostral to the clivus, surrounding the *sela turcica* and pituitary gland, and that the spongy mass of Beauregard is in fact a novel cetacean structure that represents a complex of interconnected veins between the ventral petrosal sinus, peribullar venous plexus, and pterygoid venous plexus. Although our search was not exhaustive, we have found no homologous structure in domestic mammals. The cavernous sinuses of *Tursiops* are directly connected to the middle meningeal veins, the ophthalmic plexuses, the ventral petrosal sinuses, pterygoid plexuses, and the emissary veins of the foramina ovalia (emissary vein of foramen lacerum medium of Boenninghaus). The two lateral components are connected across the midline via structures much like the intercarvernous sinuses of domestic mammals. This venous loop is therefore consistent in location and drainage with the cavernous sinus of terrestrial mammals, and seems contradictory to the statements of Fraser and Purves (1960), as it is located well within the calvarium.

An additional source of confusion arising from the descriptions of Fraser and Purves (1960) was found in their statement that “under the hydrostatic pressures available, the *corpus* (*cavernosum*) could be erected by way of the internal carotid…” This appears to conflict with our findings as well as Boenninghaus’ illustration that the *corpus* *cavernosum* is venous in nature, connected by ventral tributaries to the pterygoid and maxillary veins (#6, Figures 15A–D) and dorsal tributaries to the ventral petrosal sinus (#27, Figure 15D), rather than the carotid arterial system. Although an arterial component was observed, it appears modest and paled in comparison to the venous component. Additionally, the *corpus cavernosum* appeared grossly identical to the surrounding peribullar and pterygoid venous plexuses that connect directly to it. Therefore, it seems reasonable to assume that if the *corpus cavernosum* does indeed function as erectile tissue, this function can likely also be attributed to the venous plexuses surrounding it.

## Synopsis

Intriguing features of the veins in the head of the dolphin are the sheer volume and complexity they display. Indeed, the veins show similarly complex patterns throughout the dolphin body, often times occupying every available space within cavities and between tissues. In the head and neck, venous plexuses are seen investing the IMFBs and EMFBs, the cranial sinuses, ophthalmic regions, nasal passages, tracheal mucosa, surrounding the esophagus, within the epidural spaces, and inside the brain case. It is possible that the venous system has simply formed a network of collateral drainage pathways throughout the body (Harrison and Tomlinson, 1956), that can facilitate adequate drainage of blood from the central nervous system during periods of elevated pressure (e.g., Valsalva phenomenon), in order to avoid damaging pressure-sensitive nervous tissue (Monro–Kellie doctrine). Alternatively, it may be that despite the plexiform similarity, different portions of the venous system serve different purposes, whether collateral drainage, regional heterothermy, gas exchange, or some other function. For instance, the venous plexus found within the tracheal mucosa has been hypothesized to act as either a compensating mechanism for the reducing air volume during diving-related compression or as erectile tissue that modifies the deformation properties of the tracheal wall (Cozzi et al., 2005). Similarly, Fraser and Purves (1960) suggested that the role of the pterygoid plexuses was tied to the proper functioning of the pterygoid sinuses.

The predominant piece of information missing from this anatomical picture is the lack of functional physiological data on blood flow and gas characteristics in the aforementioned structures. We strongly suggest that further studies of these structures may shed light on important physiological processes previously discounted or neglected. We believe that a considerable amount of information can be garnered from further post-mortem studies, however we feel that relatively non-invasive, ethical, live cetacean experiments on functional morphology can be conducted and may be the only way to clarify some of the large data gaps that currently exist.

## Conflict of Interest Statement

The authors declare that the research was conducted in the absence of any commercial or financial relationships that could be construed as a potential conflict of interest.

## Supplementary Material

The Supplementary material for this article can be found online at: http://www.frontiersin.org/Aquatic_Physiology/10.3389/fphys.2012.00243/abstract

Supplementary Movie S1 **Three-dimensional reconstruction of a bottlenose dolphin with a contrast-enhanced venous system, showing spatial relationships of the structures discussed**. As the left lateral structures are clipped away, the medial aspect of the pterygoid region comes into view. Note that the colors that appear correspond to the same colors presented in Figure 1: pterygoid and peribullar venous plexus (green), anterior lobe venous plexus (purple), and IMFB plexus (yellow).

## Footnotes

^1^Lobes are partitions or outgrowths of a sinus.

^2^Traditionally venous branching descriptions follow a distal to proximal path representing the retrograde flow of venous blood; however for conceptual simplicity we have chosen to describe branching patterns starting at proximal main branches and progressing out to more distal branches.

^3^Fibrovenous plexus – a complex system of venous channels and connective tissue forming a plexus that extends from the connective tissue of the ear to the pterygoid region; it is mostly associated with the lining of the sinuses.
